# Medication De-escalation in Type 2 Diabetes: A Review of Evidence-Based Strategies for Combination Therapy Reduction

**DOI:** 10.2174/0118715303468215260624091738

**Published:** 2026-07-01

**Authors:** Omar Oraibi

**Affiliations:** 1 Department of Internal Medicine, College of Medicine, Jazan University, Jazan, Saudi Arabia

**Keywords:** Type 2 diabetes, glycemic control, polypharmacy, insulin, sulfonylureas, personalized medicine, SGLT2 inhibitors

## Abstract

This narrative review summarizes evidence on the topic of de-escalation of medications in patients with Type 2 Diabetes (T2D) who achieve glycemic control on combination therapy. The study reviews evidence from available clinical studies, expert opinions, and observations to assess the strategies, risks, and outcomes of de-escalating antidiabetic therapy in patients with T2D who have met their glycemic goals. It concludes that once the targets are achieved, continuing with complicated medication regimes can be even more risky without any benefit. Once glucose is controlled, the stepwise de-intensification of regimens may reduce polypharmacy and hypoglycemia. The evidence supports decreasing or discontinuing the use of insulin and sulfonylureas first because they are associated with the highest risk of hypoglycemia. Insulin remains the site of de-escalation. Sequential withdrawal is demonstrated to be safer than discontinuation of medications concurrently, with discontinuation of high-risk medications ranked first. SGLT2 inhibitors and GLP-1 receptor agonists have cardiovascular and renal protective effects, and therefore, it is important to consider their withdrawal. DPP-4 inhibitors may be among the safest agents to discontinue from a glycemic standpoint, given their glucose-dependent mechanism of action and low risk of hypoglycemia, while metformin should remain the mainstay of therapy. Medication de-escalation is more likely to be successful in patients who have been diabetic for a shorter period, have a lower baseline HbA1c, have fewer complications, and are committed to healthy lifestyle choices. Older people and patients with comorbidities need a more individualized approach and the chance to contribute to decision-making.

As there are limited high-quality trials to define best practices for de-escalation, structured monitoring, and clear criteria for re-escalation are required. In addition, any benefits of de-escalation must be weighed against the risks of withdrawing agents that provide cardiovascular or renal protection. Medication de-escalation seems feasible in patients who are motivated and have good glycemic control, and this is associated with a decreased treatment burden and lower risks. Nevertheless, additional quality trials are required to inform and normalize the pathways of de-intensification.

## 1 INTRODUCTION

Type 2 diabetes (T2D) is a chronic metabolic condition characterized by insulin resistance and progressive pancreatic beta-cell dysfunction, resulting in hyperglycemia [1]. Current management guidelines emphasize the timely initiation and intensification of antidiabetic therapy to achieve and maintain glycemic targets, typically assessed by glycated hemoglobin (HbA1c), to reduce microvascular and macrovascular complications [2-4]. The 2022 consensus report by the American Diabetes Association and the European Association for the Study of Diabetes (ADA–EASD) highlights the importance of a person-centered approach to diabetes management and recognizes that treatment de-intensification may be appropriate in selected patients [2]. In such cases, reduction or discontinuation of medications may decrease polypharmacy, treatment costs, and medication-related adverse effects [5].

Recent evidence has examined the feasibility and outcomes of de-escalation strategies in selected patients with T2D, including reductions or discontinuations of insulin and other glucose-lowering agents [5]. Treatment de-escalation refers to the reduction or withdrawal of medications after achieving and maintaining glycemic targets [5]. This emerging concept reflects a shift toward minimizing overtreatment and reducing the risks associated with polypharmacy in patients who have achieved metabolic control. De-escalation may reduce overtreatment, polypharmacy, and adverse effects in patients who have already reached their metabolic targets [5].

According to the ADA–EASD 2022 consensus, de-intensification of therapy may be considered when glycemic targets are achieved [2]. Potential strategies include reducing or discontinuing insulin, sulfonylureas, thiazolidinediones, SGLT2 inhibitors, GLP-1 receptor agonists, and DPP-4 inhibitors, either individually or in combination [5, 6]. This approach represents a shift from traditional glucose-centered management toward a broader model that incorporates treatment burden and patient quality of life [7, 8]. De-escalation may be beneficial in appropriately selected individuals by reducing medication-related adverse effects, treatment complexity, and costs after glycemic targets have been achieved and sustained [5].

The purpose of this narrative review is to summarize and synthesize the available evidence on strategies for de-intensification of antidiabetic medications in patients with T2D who have achieved glycemic control. This review examines approaches to reducing or discontinuing insulin, injectable therapies, and oral agents, and evaluates their effects on glycemic control, hypoglycemia, patient-reported outcomes, and healthcare costs. It also discusses criteria for patient selection, monitoring strategies, and the clinical implications of de-escalation.

## 2 LITERATURE SEARCH STRATEGY

A literature search was performed across PubMed, Embase, and Cochrane databases from database inception to May 2025. A combination of relevant keywords and index terms was used, including “diabetes,” “de-intensification,” and “deprescribing.” Specific medication names and classes were also searched for, as were appropriate synonyms. The reference lists of the returned studies and reviews were also separately searched to identify additional eligible articles.

## 3 SELECTION CRITERIA

This study included randomized controlled trials, observational studies, and systematic reviews that examined the de-escalation or discontinuation of antidiabetic medications in adults with T2D. Relevant studies reported on glycemic control (HbA1c), hypoglycemia, adverse events, clinical outcomes, medication and other treatment costs, and/or patient-reported outcomes. Studies that focused solely on medication substitution without de-intensification were excluded, and only the de-escalation intervention arms of randomized trials were summarized. Given the limited evidence, all study designs were included, without any restrictions on sample size, follow-up duration, or publication status. However, where possible, priority was given to synthesizing higher-quality evidence.

Given the narrative nature of this review, a formal quantitative synthesis was not performed. Instead, evidence was synthesized qualitatively, with emphasis placed on consistency of findings across studies. Greater weight was given to higher levels of evidence, including randomized controlled trials and systematic reviews, while observational studies and expert opinions were used to support or contextualize findings where higher-level evidence was limited. When systematic reviews were included, their conclusions were interpreted in conjunction with primary studies to avoid duplication or overrepresentation of evidence.

The literature search yielded a broad body of evidence, from which articles were selected based on their relevance to medication de-escalation in type 2 diabetes. Priority was given to randomized controlled trials, systematic reviews, and consensus guidelines, supplemented by observational studies and expert opinion where higher-level evidence was limited. A total of 45 references were included in the final synthesis.

## 4 RATIONALE FOR THE DE-ESCALATION OF TYPE 2 DIABETES

Today, a wide array of antihyperglycemic agents is available, and most patients with T2D eventually require combination therapy to achieve glycemic targets [3]. However, evidence suggests that continuing complex regimens after treatment goals have been achieved may lead to polypharmacy, increased costs, and potential harm without additional benefit [5, 9]. This has prompted interest in de-intensifying antidiabetic therapy once HbA1c can be maintained at the desired level.

There are several reasons why this approach may be beneficial. First, continuing multidrug regimens after achieving glycemic control may represent overtreatment and does not align with the principle of using the lowest effective dose [5]. Second, reducing polypharmacy through planned medication withdrawal may improve patient safety, decrease adverse effects, and reduce the risk of drug–drug interactions, particularly in older adults [10, 11]. Regimen simplification may also improve treatment adherence and reduce medication burden. Third, de-intensification may provide cost savings by eliminating unnecessary or expensive medications [12].

When deciding whether to maintain or de-escalate therapy, a patient-centered approach should consider individual circumstances, preferences, cost sensitivity, and risk of polypharmacy-related harm [2]. Several studies have demonstrated that glycemic control can be maintained in selected patients undergoing medication de-escalation. No significant increase in HbA1c has been reported following medication reduction or discontinuation [5]. Other studies have similarly shown stable glycemic control in appropriately selected patients [13]. Predictors of successful de-escalation include lifestyle modification, weight reduction, shorter disease duration, and lower baseline HbA1c [14]. However, glycemic deterioration may occur in some individuals, underscoring the importance of careful patient selection and close monitoring [15]. De-intensification appears most effective in patients with moderate glycemic control on multidrug regimens, particularly when reducing agents associated with a higher risk of hypoglycemia, such as insulin and sulfonylureas [16].

## 5 APPROACHES TO THE DE-INTENSIFICATION OF THERAPY

Various strategies are available to de-escalate antidiabetic therapy in patients with T2D. These include discontinuing selected agents, reducing polypharmacy, and reducing the remaining medications. Several approaches have been described, including gradual reduction of insulin, sulfonylureas, and other glucose-lowering agents [5].

Simplification of insulin regimens is a commonly used strategy. For example, patients may be transitioned from a basal–bolus regimen to a basal-only regimen or undergo dose reduction of insulin [11]. Other approaches include reducing or discontinuing sulfonylureas to minimize the risk of hypoglycemia [17, 18], as well as discontinuing newer agents such as SGLT2 inhibitors, GLP-1 receptor agonists, and DPP-4 inhibitors in accordance with appropriate monitoring protocols [6]. In selected, carefully monitored patients, some oral medications may be continued, while others are reduced or discontinued [19].

Insulin dose reduction can be effective but requires close monitoring of glycemic control [20]. A key principle of de-escalation is the sequential, structured tapering of medications, with ongoing assessment of glycemic trends, rather than abrupt discontinuation [5, 21].

These considerations apply to patients with T2D. In contrast, de-intensification is generally not appropriate in individuals with type 1 diabetes (T1D), who require lifelong insulin replacement due to absolute insulin deficiency. Any attempt to reduce insulin in this population should be undertaken with extreme caution and close monitoring [22]. De-escalation strategies should always be individualized based on patient characteristics and clinical context.

## 6 EVIDENCE FOR DE-ESCALATION BY MEDICATION CLASS

### 6.1 Insulin De-escalation

Insulin is the most extensively studied agent in de-escalation strategies. Insulin dose reduction or discontinuation can be successfully achieved in appropriately selected patients, particularly those with preserved beta-cell function and strong adherence to lifestyle modifications [5]. Simplification of complex insulin regimens has been shown to maintain glycemic control in selected patients [11]. Evidence suggests that insulin de-escalation is most effective in patients with shorter duration of type 2 diabetes, lower baseline HbA1c, and those engaged in intensive lifestyle interventions [11]. However, careful monitoring is essential, as the risk of glycemic deterioration is highest following insulin withdrawal [5].

### 6.2 Sulfonylurea Reduction and Discontinuation

Sulfonylureas are commonly targeted for de-escalation due to their high risk of hypoglycemia. The role of sulfonylureas should be reassessed when the risk of hypoglycemia outweighs glycemic benefit [17]. Evidence indicates that sulfonylurea de-escalation is particularly beneficial in older adults, especially those with a history of hypoglycemic episodes [16]. Gradual dose reduction is generally preferred over abrupt discontinuation to minimize the risk of rebound hyperglycemia [5].

### 6.3 Newer Antidiabetic Agents

Evidence regarding the de-escalation of newer antidiabetic agents, including SGLT2 inhibitors, GLP-1 receptor agonists, and DPP-4 inhibitors, remains limited but is emerging. GLP-1 receptor agonists and DPP-4 inhibitors are associated with a lower risk of hypoglycemia [6]. Discontinuation of SGLT2 inhibitors and GLP-1 receptor agonists requires careful consideration of their cardiovascular and renal benefits [5]. DPP-4 inhibitors may be among the safest classes to discontinue from a glycemic standpoint because of their glucose-dependent mechanism of action and minimal risk of hypoglycemia, although individual patient factors should guide decision-making [5].

### 6.4 Metformin Considerations

Metformin is generally not considered a candidate for de-escalation due to its favorable safety profile and its continued role as foundational first-line therapy [23-26]. Even when other agents are reduced or discontinued, current evidence supports continuation of metformin unless contraindicated by renal impairment or intolerance [25, 26].

### 6.5 Thiazolidinediones

There is limited evidence supporting the de-escalation of thiazolidinediones. These agents may be discontinued due to their known adverse effects, including weight gain, fluid retention, and increased fracture risk, particularly in older adults [10]. Decisions to discontinue thiazolidinediones should be individualized based on patient-specific risk factors and the availability of alternative therapies to maintain glycemic control.

### 6.6 Sequential *versus* Simultaneous De-escalation

Evidence suggests that sequential drug withdrawal is safer than the simultaneous discontinuation of multiple agents. De-escalation should initially target medications associated with the highest risk of hypoglycemia, particularly insulin and sulfonylureas [27]. In carefully selected patients with stable glycemic control and a strong commitment to lifestyle modification, reducing multiple agents may be considered; however, this approach requires close monitoring and structured follow-up [28, 29].

### 6.7 Class-Specific Success Predictors

For insulin de-escalation, preserved β-cell function-often assessed by C-peptide levels-is associated with a greater likelihood of maintaining glycemic control after dose reduction [29]. In addition, overall medication burden and the potential effect of polypharmacy on healthcare utilization should be considered when planning de-escalation in older adults with comorbidities [30]. For sulfonylureas, a lower frequency of hypoglycemic episodes and preserved beta-cell function are important considerations; glycemic targets and risk–benefit balance should also be individualized [31]. For newer agents, key factors include the availability of alternative mechanisms for glycemic control and the absence of established cardiovascular indications [32].

A summary of the evidence for de-escalation by antidiabetic medication class, including evidence levels, outcomes, predictors of success, and associated risks, is presented in Table **1**.

### 6.8 De-escalation in Special Populations

Special attention should be given to de-intensification in older adults, patients with comorbidities, and those at increased risk of hypoglycemia. Older adults are particularly vulnerable to the adverse effects of polypharmacy and may benefit from treatment simplification [33]. Hypoglycemia risk, life expectancy, and functional status should be considered when individualizing therapy in older adults [16]. Additional age-related factors influencing treatment decisions include frailty, cognitive impairment, and reduced renal function [33, 34].

Glycemic management in patients with Chronic Kidney Disease (CKD) requires careful consideration [15]. De-escalation may also be appropriate in patients with significant cardiac, hepatic, or renal comorbidities, provided that close monitoring is maintained [33, 34]. Reduction or discontinuation of insulin and sulfonylureas appears particularly beneficial in patients with a history of hypoglycemia or hypoglycemia unawareness, when alternative therapies can maintain adequate glycemic control [35].

Injectable agents may help address hypoglycemia risk and overcome clinical inertia in selected older adults [32]. Clinical judgment that carefully balances risks and benefits, along with consideration of patient-specific factors and regimen complexity, is essential when making de-intensification decisions in this vulnerable population [33]. Despite potential benefits, de-escalation strategies should be implemented cautiously and require close follow-up in high-risk individuals [33, 34].

## 7 RISKS OF DE-ESCALATION IN MODERATE- AND HIGH-RISK PATIENTS

De-escalation of antidiabetic therapy, while beneficial in selected patients, carries important risks when applied to individuals with moderate to high cardiometabolic risk. One of the primary concerns is deterioration of glycemic control, which may lead to symptomatic hyperglycemia and, in some cases, acute metabolic complications if not promptly identified and managed [5, 15, 35, 36]. Patients with longer disease duration, reduced β-cell function, or higher baseline HbA1c are particularly vulnerable to glycemic relapse following therapy reduction [5, 15].

In addition to glycemic deterioration, discontinuation of certain antidiabetic agents may result in loss of established cardiovascular and renal protective benefits, particularly those demonstrated in large cardiovascular outcomes trials [37-39]. This is particularly relevant for SGLT2 inhibitors and GLP-1 receptor agonists, which have demonstrated outcome benefits independent of glycemic control [2, 7, 40, 41]. In high-risk patients, inappropriate withdrawal of these agents may adversely affect long-term cardiovascular and renal outcomes, as demonstrated in major randomized trials of SGLT2 inhibitors and GLP-1 receptor agonists [37-39], and current guidelines generally support their continuation when clinically indicated [2, 36].

Another important consideration is the potential for therapeutic inertia following de-escalation, a well-recognized phenomenon in diabetes care that may delay appropriate re-intensification [21, 35, 42]. Delayed re-intensification of therapy in the setting of worsening glycemic control may expose patients to prolonged periods of hyperglycemia, thereby increasing the risk of diabetes-related complications [35, 42]. In addition, inadequate monitoring after medication reduction may delay recognition of glycemic deterioration, further compounding this risk [35].

Older adults, patients with CKD, and those with a history of hypoglycemia require particularly careful, individualized decision-making, as both overtreatment and undertreatment may result in adverse outcomes [15, 16, 43]. Therefore, de-escalation in moderate- and high-risk patients should be undertaken cautiously, with individualized risk–benefit assessment, close monitoring, and clearly defined criteria for re-escalation [2, 35, 36].

## 8 INTERACTION WITH OTHER CARDIOMETABOLIC THERAPIESA

De-escalation of antidiabetic therapy should be considered within the broader context of comprehensive cardiometabolic management, including the use of lipid-lowering and antihypertensive medications. These therapies play a critical role in reducing cardiovascular risk and may influence treatment priorities when adjusting antidiabetic regimens [2, 7]. In patients with established cardiovascular disease or high cardiometabolic risk, lipid-lowering therapies such as statins and antihypertensive agents-including renin–angiotensin system inhibitors-are often prioritized for long-term risk reduction in accordance with contemporary cardiovascular guidelines [44].

The presence of effective cardiovascular risk control may influence glycemic targets and the overall therapeutic strategy. In patients receiving comprehensive cardio- metabolic management, clinicians may adopt a more individualized and less intensive glycemic approach, allowing cautious de-escalation of glucose-lowering medications while maintaining strict control of blood pressure and lipid levels [2, 35]. Conversely, in patients with persistent or high cardiovascular risk, maintaining antidiabetic agents with proven cardioprotective and renoprotective benefits may take precedence over simplification of therapy [2, 7, 37-39].

In addition, certain antihypertensive agents may influence glycemic control or the recognition of hypoglycemia. For example, beta-blockers may mask adrenergic symptoms of hypoglycemia, while thiazide diuretics may adversely affect glucose metabolism. These interactions should be considered when planning de-escalation strategies and monitoring glycemic trends following therapy adjustment [15, 20].

Therefore, de-escalation decisions should be made within the context of an integrated, multidisciplinary approach that considers the cumulative metabolic effects of all therapies, ensuring that reductions in glucose-lowering treatment do not compromise overall cardiovascular or renal risk reduction [2].

## 9 PATIENT PREFERENCES AND SHARED DECISION-MAKING

Patient motivations for de-intensification of antidiabetic regimens vary. Some patients may wish to reduce medication burden after achieving glycemic control. Common reasons include minimizing side effects, reducing treatment burden, avoiding injectable therapies, addressing cost concerns, reducing the risk of hypoglycemia, and exploring lifestyle-based management before reintroducing medications [32]. Barriers associated with injectable therapies in older adults further underscore the importance of incorporating patient preferences into treatment decisions [32].

Understanding patient experiences, preferences, and values is central to successful de-escalation. Effective de-escalation requires patient engagement and shared decision-making to ensure sustained glycemic control [2, 33]. Shared decision-making helps ensure that de-intensification strategies remain patient-centered [33].

Providers and patients should engage in open discussions regarding the risks and benefits of reducing treatment intensity in the context of individual goals and priorities [5]. Clinicians should assess patient preferences regarding acceptable glycemic targets, medication choices, and willingness to re-escalate therapy if needed [2].

Patients undergoing de-escalation should receive appropriate education and close monitoring, with predefined action plans in place to address potential glycemic deterioration [2, 34].

## 10 MONITORING GLYCEMIC CONTROL AND RE-ESCALATION

Regular follow-up and timely re-intensification are essential components of a de-escalation strategy to prevent prolonged periods of hyperglycemia [2]. A structured SMBG approach has been recommended, including testing 1–2 times daily after treatment reduction and HbA1c assessment every 3 months [35]. More frequent SMBG, including pre- and postprandial measurements, is recommended for patients using insulin who are at higher risk of hypoglycemia [35].

Clear, written action plans should be provided to guide patients on when to contact healthcare providers, for example, if hyperglycemia persists above 200 mg/dL for more than 3 days [20]. Explicit re-escalation criteria are essential. ADA Standards of Care recommend returning to prior therapy if HbA1c rises more than 0.5% above the individualized target or if hyperglycemic symptoms persist [35]. Prompt re-escalation is preferred to avoid prolonged uncontrolled hyperglycemia and long-term metabolic deterioration [2, 35, 45].

A proactive, responsive approach allows timely re-intensification without unnecessary overtreatment.

Overall, de-escalation requires proactive follow-up and continuous communication between patients and healthcare providers. With close monitoring and predefined re-escalation protocols, episodes of hyperglycemia associated with de-escalation can be minimized [20].

## 11 FUTURE DIRECTIONS OF STUDY

Studies have demonstrated the feasibility of de-intensification in selected populations with T2D; however, further research is needed to refine its implementation.

Key areas for future investigation include identifying clinical and biomarker predictors beyond HbA1c that can reliably predict sustained glycemic control after medication de-escalation. Comparative studies evaluating different pharmacologic agents and tapering strategies are also needed to determine the safest and most effective approaches. In addition, patient-reported outcomes-including treatment satisfaction, quality of life, and emotional well-being-should be further explored, as they are central to patient-centered care.

Long-term data on clinical outcomes and diabetes-related complications following de-intensification remain limited, as most existing studies provide only short- to medium-term follow-up [13]. Cost-effectiveness analyses are also needed to better understand the economic impact of de-escalation strategies on healthcare systems [10, 12].

Standardized protocols and clinician training are needed to support lifestyle-based de-escalation strategies [22]. Advances in digital health tools and continuous glucose monitoring may further enhance monitoring during de-escalation. Future research should also focus on identifying biomarkers or genetic factors that predict successful medication reduction [5].

Additional research gaps include determining the optimal timing for initiating de-escalation, evaluating the role of diabetes technologies in supporting medication reduction, and assessing the long-term effects on cardiovascular and microvascular outcomes [5, 15]. Studies across diverse healthcare settings and populations, including different ethnic groups, are necessary to establish the generalizability of these strategies [5, 15].

Future research should also focus on clearly defined and actionable questions to guide clinical implementation. Key priorities include identifying reliable biomarkers (such as C-peptide or other indicators of β-cell function) that can predict successful de-escalation, developing continuous glucose monitoring (CGM)-guided protocols to safely adjust therapy, and establishing standardized criteria for initiating and reversing de-intensification. In addition, prospective studies comparing different de-escalation strategies across medication classes and patient populations are needed to define optimal sequencing and monitoring approaches in routine clinical practice.

## CONCLUSION

De-escalation of antidiabetic therapy represents an important component of individualized diabetes management in selected patients with type 2 diabetes who achieve stable glycemic control. Evidence supports a structured, stepwise reduction of therapy, particularly targeting agents associated with higher hypoglycemia risk, while preserving medications with demonstrated cardiovascular and renal benefits.

Successful de-escalation requires careful patient selection, shared decision-making, and ongoing monitoring to maintain glycemic targets and promptly re-escalate when necessary. Special consideration should be given to patients with higher cardiometabolic risk, in whom inappropriate de-intensification may lead to adverse outcomes.

Further high-quality studies are needed to define optimal strategies, identify predictors of success, and develop standardized protocols to guide safe and effective de-escalation in clinical practice.

## AUTHORS’ CONTRIBUTIONS

The author confirms sole responsibility for the following: study conception and design, data collection, analysis and interpretation of results, and manuscript preparation.

## Figures and Tables

**Table 1:** Summary of evidence for de-escalation by antidiabetic medication class.

**Medication Class**	**Evidence Level**	**Main Outcomes After De-escalation**	**Predictors of Success**	**Risks of De-escalation**
Insulin	Moderate (observational studies and small trials)	Maintenance of HbA1c in selected patients; reduced hypoglycemia; simplification of treatment regimens (*e.g*., basal-only)	Shorter T2D duration; lower baseline HbA1c; preserved β- cell function; strong adherence to lifestyle modifications	Highest risk of glycemic deterioration; symptomatic hyperglycemia
Sulfonylureas	Moderate (observational studies and clinical data)	Reduced frequency of hypoglycemia, particularly in older adults; minimal change in HbA1c when gradually reduced or discontinued	Absence of recent hypoglycemia; adequate β-cell reserve	Rebound hyperglycemia if discontinued abruptly
SGLT2 Inhibitors	Low to moderate (emerging clinical data)	Maintenance of glycemic control in selected patients; potential loss of cardiovascular and renal protective effects upon discontinuation	Absence of cardiovascular or renal indications; stable glycemic control	Worsening of heart failure or renal outcomes in high-risk patients
GLP-1 Receptor Agonists	Low to moderate (emerging clinical data)	Maintenance of glycemic control in selected patients; possible weight regain after discontinuation	Stable HbA1c without dependence on weight reduction benefits	Loss of cardiovascular benefit; weight gain; reduced satiety
DPP-4 Inhibitors	Low (limited evidence)	Minimal impact on glycemic control when discontinued in well-controlled patients	Presence of alternative glycemic control mechanisms	Mild increase in HbA1c
Metformin	strong (consistent evidence from RCTs and guidelines)	Sustained glycemic control; cardiovascular benefit; weight neutrality	Not applicable; generally recommended to continue unless contraindicated	Loss of glycemic control and cardiovascular benefit if discontinued
Thiazolidinediones (TZDs)	Low (limited evidence)	Reduction in adverse effects such as weight gain, fluid retention, and fracture risk	Availability of alternative therapies; absence of heart failure	Deterioration of glycemic control if therapy-dependent

## LIST OF ABBREVIATIONS

ADA: American Diabetes Association
EASD: European Association for the Study of Diabetes
T2D: Type 2 Diabetes
T1D: Type 1 Diabetes
HbA1c: Glycated Hemoglobin
CKD: Chronic Kidney Disease
SMBG: Self-Monitoring of Blood Glucose
SGLT2: Sodium–Glucose Cotransporter 2
GLP-1: Glucagon-Like Peptide-1
DPP-4: Dipeptidyl Peptidase-4
TZDs: Thiazolidinediones
CGM: Continuous Glucose Monitoring
RCTs: Randomized Controlled Trials
